# Physiological Adaptation Strategy of the Pseudo-Metallophyte *Lotus corniculatus* L. to Long-Term Metal Contamination

**DOI:** 10.3390/antiox15080969

**Published:** 2026-08-05

**Authors:** Marzena Sujkowska-Rybkowska, Anna Rusaczonek, Małgorzata Nykiel, Ewelina Hallmann, Maria Duszyn, Sławomir Jaworski, Wojciech Borucki

**Affiliations:** 1 Department of Botany and Plant Physiology, Institute of Biology, Warsaw University of Life Sciences—SGGW, Nowoursynowska 159, 02-776 Warsaw, Poland; anna_rusaczonek@sggw.edu.pl (A.R.); wojciech_borucki@sggw.edu.pl (W.B.); 2 Department of Biochemistry and Microbiology, Institute of Biology, Warsaw University of Life Sciences—SGGW, Nowoursynowska 159, 02-776 Warsaw, Poland; malgorzata_nykiel@sggw.edu.pl; 3 Department of Functional and Organic Food, Institute of Human Nutrition Sciences, Warsaw University of Life Sciences—SGGW, Nowoursynowska 159, 02-776 Warsaw, Poland; ewelina_hallmann@sggw.edu.pl; 4 Bioeconomy Research Institute, Agriculture Academy, Vytautas Magnus University, Donelaicio 58, 44248 Kaunas, Lithuania; 5 Department of Plant Genetics, Breeding and Biotechnology, Institute of Biology, Warsaw University of Life Sciences—SGGW, Nowoursynowska 159, 02-776 Warsaw, Poland; maria_duszyn@sggw.edu.pl; 6 Department of Nanobiotechnology, Institute of Biology, Warsaw University of Life Sciences—SGGW, 02-776 Warsaw, Poland; slawomir_jaworski@sggw.edu.pl

**Keywords:** antioxidant, calamine tailings, *Lotus corniculatus*, heavy metal stress, physiological characteristics

## Abstract

*Lotus corniculatus* L. can spontaneously colonize metal-contaminated areas like the old Zn-Pb calamine tailings. This study compared two *L. corniculatus* ecotypes collected from metal-contaminated (HM) and non-contaminated (NM) sites to identify physiological adaptations to long-term metal exposure. The results indicated that HM ecotype accumulates Zn, Pb, and Cd in shoots without showing any toxic symptoms at the ultrastructural level. The HM ecotype exhibited a different composition of photosynthetic pigments, lower lipid peroxidation, and oxidative stress levels and increased the efficiency of the antioxidant defense system and antioxidant activity compared to the NM ecotype. Leaf antioxidant enzymes such as SOD, CAT, POX, and GPX were more active in the shoots of the HM ecotype, while GR and APX showed lower activity compared to the NM ecotype. In addition, the HM ecotype was characterized by higher glutathione and total phenolics content (mainly catechin, rutin, ferulic, and salicylic acids); however, these non-enzymatic metabolites primarily function in metal chelation alongside their role in oxidative stress mitigation. The results showed that calamine *Lotus* plants exhibit effective adaptation to long-term metal exposure by keeping metals away from metabolically active compartments and strengthening antioxidant defenses. These coordinated mechanisms reduce oxidative stress and contribute to the enhanced metal tolerance of calamine plants.

## 1. Introduction

Heavy metal contamination represents one of the most widespread problems resulting from their bioaccumulation in living organisms, their high toxicity, and their non-biodegradability [1]. The landscape of southern Poland is rich in metal-bearing heaps, as a result of metal ore extraction and processing in this area since the 13th century. The 100-year-old Zn-Pb calamine waste sites near Olkusz are heavily metal-contaminated with harsh ecological conditions, such as nutrient and water deficiencies, high insolation, and strong winds [2,3,4,5]. These harsh conditions have driven the evolution of genetic and physiological adaptations that enable organisms to colonize and persist in these extreme environments [6]. Metal-tolerant legumes are of scientific interest owing to their high adaptation capacity and tolerance to heavy metals, with potential use in agricultural and ecological contexts [7]. It is postulated that their adaptation success results from their ability to engage in symbiotic interactions with nitrogen-fixing rhizobia and arbuscular mycorrhizal fungi that facilitate phosphorus uptake, even in metal-bearing sites [2,3,4,5]. This in turn relates to the ability of these organisms to support plant metal adaptation by improving physiological functions [2,5,8,9]. While research on root-metal symbioses has been conducted in several studies [2,4,5,10], the examination of adaptive structural, physiological, or molecular changes in the above-ground parts of legumes growing on metal-bearing wastes is rare.

The accumulation of toxic metals in plant tissues is a significant risk to plant growth and development, leading to oxidative stress associated with the increased generation of reactive oxygen species (ROS) [11]. It is well documented that exposure to heavy metals can affect photosynthesis and reduce the photosynthetic pigment content [12]. The plant’s antioxidant defense system usually controls the overproduction of ROS under heavy metal stress. Metal-tolerant plants have developed these systems using non-enzymatic components of the antioxidant defense system, including ascorbic acid (AsA), glutathione (GSH), carotenoids, and polyphenolic compounds, as well as enzymatic antioxidants based on enzymes such as superoxide dismutase (SOD), peroxidase (POX), catalase (CAT), and glutathione-ascorbate cycle enzymes (glutathione peroxidase (GPX), ascorbate peroxidase (APX), guaiacol peroxidase (GPX), monodehydroascorbate reductase (MDHAR), and glutathione reductase (GR)). These elements have been most frequently analyzed in studies on plant antioxidant defense responses to heavy metals and xenobiotics [13,14]. The non-enzymatic antioxidants found in plants are mainly determined by measuring antioxidant activity, which is typically determined using complementary assays, including DPPH (1,1-diphenyl-2-picrylhydrazy) radical scavenging, ferric reducing antioxidant power (FRAP), and/or ABTS (2,2-azinobis-ethylbenzothiozoline-6-sulphonic acid) radical cation decolorization methods [15].

In addition to scavenging radicals, non-enzymatic antioxidants (mainly GSH and polyphenols) also act as metal chelators [14,16,17]. GSH contributes to heavy metal detoxification by acting as a substrate for glutathione S-transferases and as the biosynthetic precursor of phytochelatins [18]. In some plants, such as legumes, homoglutathione (glutamyl cysteinyl alanine), the homolog of GSH, may partly or wholly replace glutathione. Root tissues contained higher levels of homoglutathione, while GSH predominated in leaves and seeds of legume plants [19]. Phenolic compounds also show the ability to form complexes with different metal ions [16,20]. Phenolic acids, like protocatechuic acid, vanillic acid, gallic acid, syringic acid, cinnamic acid, p-coumaric acid, chlorogenic acid, and caffeic acid, show strong ability for metal complexation. The strongest metal-chelating activity among flavonols has been attributed to quercetin and its glycosylated derivatives [16,20].

*Lotus corniculatus* L. (birdsfoot trefoil) is one of the legume species that has achieved adaptation success and spontaneously colonizes the old calamine tailings [2,4]. This species exhibits greater adaptability potential compared to other *Lotus* varieties, contributing to its rapid growth, high metal tolerance, and extensive root system. Consequently, it has become a preferred species for different phytoremediation projects [21]. Nevertheless, systematic studies on this species’ adaptation abilities for growth and development on metal-contaminated sites remain limited. The present study focused on comparing the physiological responses of birdsfoot trefoil plants growing in metal-contaminated tailings with those from non-polluted areas. We analyzed (1) the accumulation of HMs in tissues and their direct impact on photosynthetic efficiency by observing changes in chloroplast structure and pigments composition and (2) the characterization of defense mechanisms through the analysis of antioxidant enzyme activities and non-enzymatic antioxidant-mediated protection against metal-induced oxidative stress. Overall, this work contributes to a better understanding of the mechanisms by which selected ecotypes minimize metal toxicity in their environment. The findings expand current knowledge of the remarkable adaptability of calamine flora to extreme conditions.

## 2. Materials and Methods

### 2.1. The Experimental Material

Two ecotypes of *L. corniculatus*—a non-metallicolous ecotype (referred to as NM) originating from a stonepit in Kazimierz Dolny (51°19′ N 21°56′ E) and the metallicolous ecotype (referred to as HM) collected from the old calamine tailing in Bolesław (50°17′ N 19°29′ E)—were sampled according to our published protocol [2,3]. The soil analysis showed that the total concentrations of Zn, Pb, and Cd in the calamine substrate reached 42.983, 2297.9, and 183.6 mg kg^−1^, respectively, whereas the corresponding concentrations in the NM soil were 55.6, 10.4, and 1.0 mg kg^−1^ [3].

### 2.2. Concentration of Metals in Plants

Zn, Pb, and Cd concentrations in plant tissues were determined using inductively coupled plasma mass spectrometry (ICP-MS) following the procedure described previously [3]. Metal contents were measured in the shoots and roots of both ecotypes, and translocation factors (TFs) were calculated for each metal using the formula TF = Metal concentration in shoots/Metal concentration in roots.

### 2.3. Chloroplasts Ultrastructure

Nine fully expanded leaves from each ecotype were fixed in Karnovsky’s medium, embedded in epoxy resin, and processed for ultrastructural examination by transmission electron microscopy (TEM), as previously described [2]. The prepared grids with leaf samples were analyzed using TEM (FEI 268D ‘Morgagni’ FEI Company, Hillsboro, OR, USA). Next, the photographic records of the observed chloroplasts were produced.

### 2.4. Photosynthetic Pigments Content and De-Epoxidation State

Pigments were extracted from frozen tissue with cold acetone and analyzed by HPLC according to the method described previously [22]. Pigments were identified using authentic standards and quantified as peak area per μg fresh weight. Total carotenoids were calculated as the sum of xanthophylls and carotenes, and the de-epoxidation state (DES) was determined as (A/2 + Z)/(Z + A + V), where Z is zeaxanthin, V is violaxanthin, and A is antheraxanthin, respectively.

### 2.5. Patterns of ROS Distribution, Cell Death, and Lipid Peroxidation

For the histochemical detection of H_2_O_2_, the leaves were infiltrated with diaminobenzidine (DAB) according to Daudi et al. [23]. The presence of superoxide (O_2_^•−^) in leaves was detected with nitroblue tetrazolium (NBT) according to our earlier study [3]. Cell death was visualized by 1% (*m*/*v*) Evans blue staining, followed by chlorophyll clearing and imaging under a stereomicroscope. Hydrogen peroxide localization was also performed by TEM using CeCl_3_, as previously described [3].

Lipid peroxidation was assessed by measuring MDA (malondialdehyde) content according to Hodges et al. [24]. The MDA concentration was determined spectrophotometrically and expressed as μg g^−1^ fresh weight (FW).

### 2.6. Antioxidant Activities

The antioxidant capacity of methanolic extracts was assessed using ABTS, DPPH, and FRAP assays. Freeze-dried tissue extracts were analyzed according to Re et al. [25], the original DPPH protocol [26], and the FRAP method [27]. ABTS and DPPH activities were expressed as percentage inhibition, while FRAP activity was calculated as percentage reduction relative to ascorbic acid.

### 2.7. Phenolics (Phs) and Chlorophyll (Chl) Autofluorescence with Confocal Laser Scanning Microscopy (CLSM)

*Phs* and *Chl* autofluorescence was examined on the hand cross-sections through the leaf blade of Lotus plants. Fresh leaf blade sections were examined using a Leica TCS SP5II confocal laser scanning microscope. Chlorophyll and phenolic compound autofluorescence were excited at 405 nm and recorded at 420–500 and 670–730 nm, respectively.

The emission spectra of phenolics were examined using Lambda scanning (after excitation with a 405 nm wavelength) to show changes in the fluorescence emission spectra of vacuolar phenolics in the leaf mesophyll of NM and HM plants. The lambda scan was performed from 420 to 620 nm in 46 steps and a 10 nm bandwidth, always under the same experimental settings.

### 2.8. Total Phenolic Content

The total polyphenol content was measured with the use of the Folin–Ciocâlteu reagent according to [28]. Methanolic extracts were analyzed spectrophotometrically at 765 nm and expressed as mg gallic acid equivalents (GAE) per g fresh weight (FW).

### 2.9. Polyphenol Profile with HPLC Analysis

Polyphenols were extracted from fresh leaf tissue using 80% methanol and quantified by HPLC [Shimadzu system (Tokyo, Japan)] using a Phenomenex Fusion-RP column, as described in a previous study [29]. Compounds were identified and quantified using external standards based on retention times and calibration curves [29].

### 2.10. Glutathione Content and Localization

Reduced (GSH) and oxidized glutathione (GSSG) were determined by enzymatic recycling assay according to [30], with absorbance measured at 412 nm. GSH content was calculated from total glutathione and GSSG levels.

Histochemical GSH localization in *Lotus* leaves was carried out with monochlorobimane (MCB) (Molecular Probes Inc., Eugene, OR, USA) as a fluorescent marker [31], where MCB gives glutathione S-bimane (GSB) conjugates. GSB and chlorophyll fluorescence were analyzed by confocal microscopy (Leica TCS SP5 II, Leica Microsystems, Wetzlar, Germany) after excitation at 405 nm.

### 2.11. Ascorbate Content

Ascorbate (AsA) content was determined spectrophotometrically according to [32] using leaf extracts prepared in 0.1 M HCl and neutralized with phosphate buffer. Total AsA was determined after the reduction of dehydroascorbate with 10 mM DTT, followed by N-ethylmaleimide treatment, whereas untreated aliquots were used for reduced ascorbate determination. Samples were precipitated with 10% TCA, centrifuged, and reacted with 2,2′-bipyridine/FeCl_3_ reagent. Absorbance was measured at 525 nm, and the ascorbate concentration was calculated from a standard curve.

### 2.12. Antioxidant Enzymes Activity Measurements

For enzyme activity assays, collected leaf samples (100 mg) were frozen in liquid nitrogen and maintained at −80 °C until protein extraction. The spectrophotometric assays of superoxide dismutase (SOD; EC 1.15.1.1) and catalase (CAT; EC 1.11.1.6) activities were conducted in accordance with methods [3,33].

The cytochemical localization of CAT and POX was performed according to [3]. Epoxy resin-embedded leaf sections (3 µm) were analyzed using light microscopy (Olympus AX70 Provis, Olympus, Tokyo, Japan).

Ascorbate peroxidase (APX; EC 1.11.1.11), monodehydroascorbate reductase (MDAR; EC 1.6.5.4), glutathione peroxidase (GPX; EC 1.11.1.9), and glutathione reductase (GR; EC 1.6.4.2) activities were determined spectrophotometrically according to procedures [34,35,36] using leaf protein extracts. Enzyme activities were calculated from substrate oxidation or product formation and expressed per minute per mg of protein.

### 2.13. Statistical Analysis

Statistical analyses were performed using STATISTICA 13.0 (StatSoft, Inc., Tulsa, OK, USA). Differences among treatments were evaluated by one-way ANOVA followed by Tukey’s HSD test at *p* < 0.05. For heatmap visualization, data were scaled relative to the mean value of both ecotypes for each parameter, with the mean set to 100. The heatmap was generated using GraphPad Prism version 8.0 software (Dot-matics, San Diego, CA, USA).

## 3. Results

### 3.1. Metal Content in Plants

Despite elevated concentrations of mobile Pb, Cd, and Zn in the calamine substratum compared with the mean values reported for Poland and Central Europe [2], the HM ecotype mainly retained HMs in the roots according to ICP-MS analysis (Table 1). The translocation factor (TF) is a widely used index to evaluate the ability of plants to transport metals from roots to shoots. TF above 1 indicates efficient root-to-shoot transport and is typical for many hyperaccumulators. Our analysis confirms that this species does not hyperaccumulate metals, as the average value of the translocation factor was below 1, which is typical for metal excluders. In the case of metal accumulation in roots, the HM ecotype represented about a 50-fold-higher concentration of Zn, a 23-fold-higher content of Pb, and a 70-fold-higher concentration of Cd than the reference NM ecotype. In the case of shoots, the HM ecotype showed about a 19-fold-higher concentration of Zn, a 7.5-fold-higher content of Pb, and about a 70-fold-higher concentration of Cd than the reference NM ecotype (Table 1).

### 3.2. Ultrastructure and Accumulation of Photosynthetic Pigments in Chloroplasts

**Figure 1:**
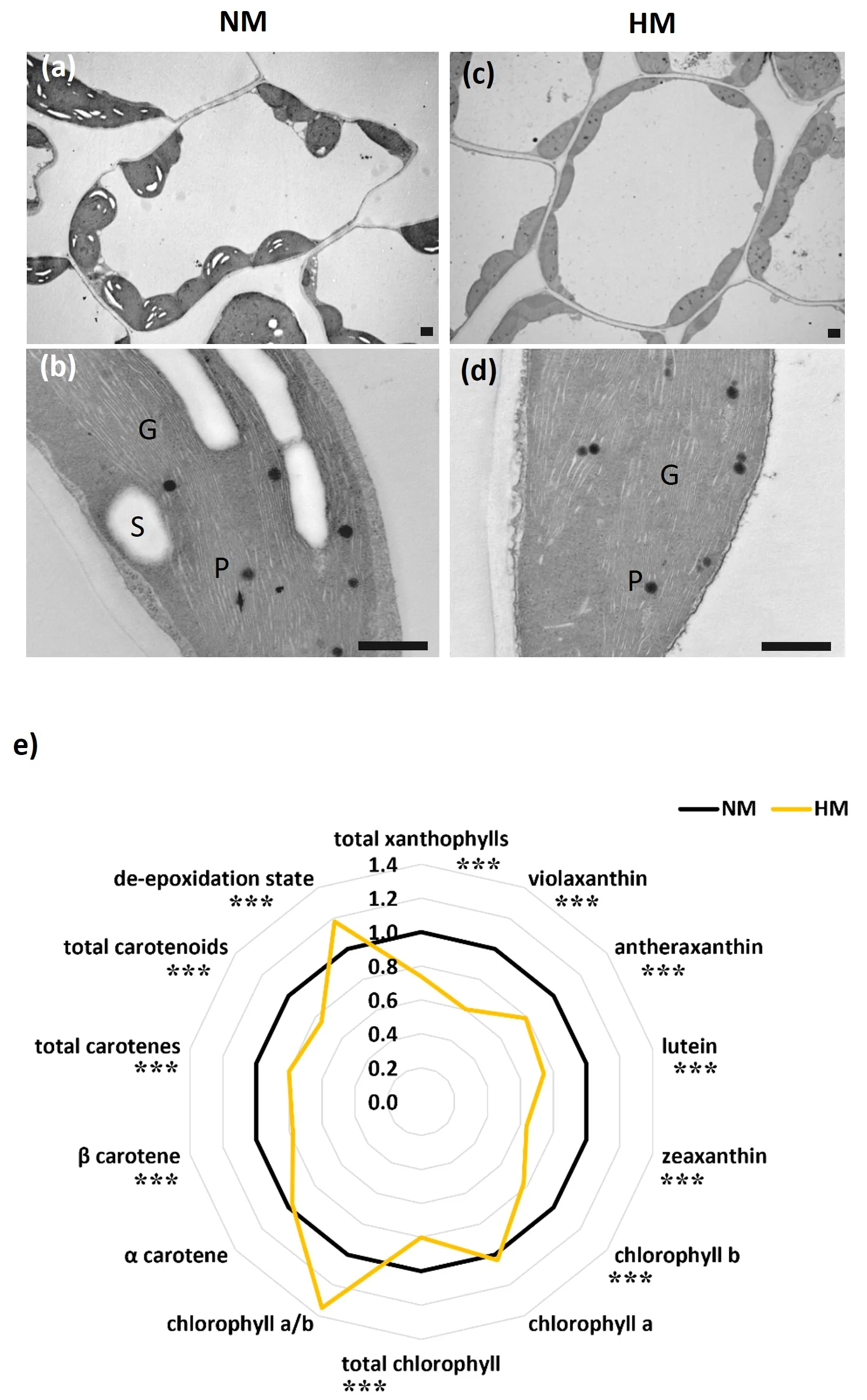
Mesophyll cells (**a**,**c**) and magnified chloroplasts (**b**,**d**) in leaves of NM and HM Lotus ecotypes. Abbreviations: G—granum, P—plastoglobule, S—starch. Bars = 1 µm. (**e**) Spider-plot of selected photosynthetic pigments in NM and HM ecotypes calculated from the induction curve normalized to the NM. Data represent mean values from at least six plants (*n* ≥ 6). The stars above the bars indicate significant differences significant differences (one-way ANOVA, Tukey’s test, *p* < 0.05).

Owing to the high HM content in shoots of the HM ecotype, the chloroplast structure and photosynthetic pigment composition were examined. Leaf ultrastructure analysis showed that chloroplasts of both ecotypes retained a regular structure with well-developed membranes, grana, and a few small plastoglobules, suggesting no detrimental effects of metals on chloroplast organization. However, in the chloroplasts of the HM ecotype, starch grains were not detected (Figure 1a–d).

We found that L. corniculatus ecotypes differ in the content and composition of photosynthetic pigments (Figure 1e). Statistical analysis using one-way ANOVA demonstrated significant differences in HM shoots, with a significantly lower content of most photosynthetic pigments in comparison with the reference NM shoots (Figure 1e). In HM shoots, a lower total chlorophyll content was observed, resulting from a decrease in chl *b*, while chl *a* was in similar amounts in both ecotypes. The chlorophyll a/b ratio was higher in HM, but this increase was not significant.

The HM ecotype showed significantly lower levels of total carotenoids, xanthophylls, and carotenes. The content of xanthophyll pigments, i.e., antheraxanthin, violaxanthin, zeaxanthin, and lutein, showed significantly lower content in HM shoots. In HM shoots, the reduced content of total carotenes resulted from the lower content of beta-carotene, as alpha-carotene did not differ between the samples (Figure 1e).

The de-epoxidation state of xanthophyll cycle pigments, considered as a key indicator of plants’ photoprotection ability against excess light, reaches significantly higher values in the HM ecotype compared to the NM (Figure 1e).

### 3.3. ROS Production, Oxidative Stress Intensity, and Antioxidant Defense Capacity

**Figure 2:**
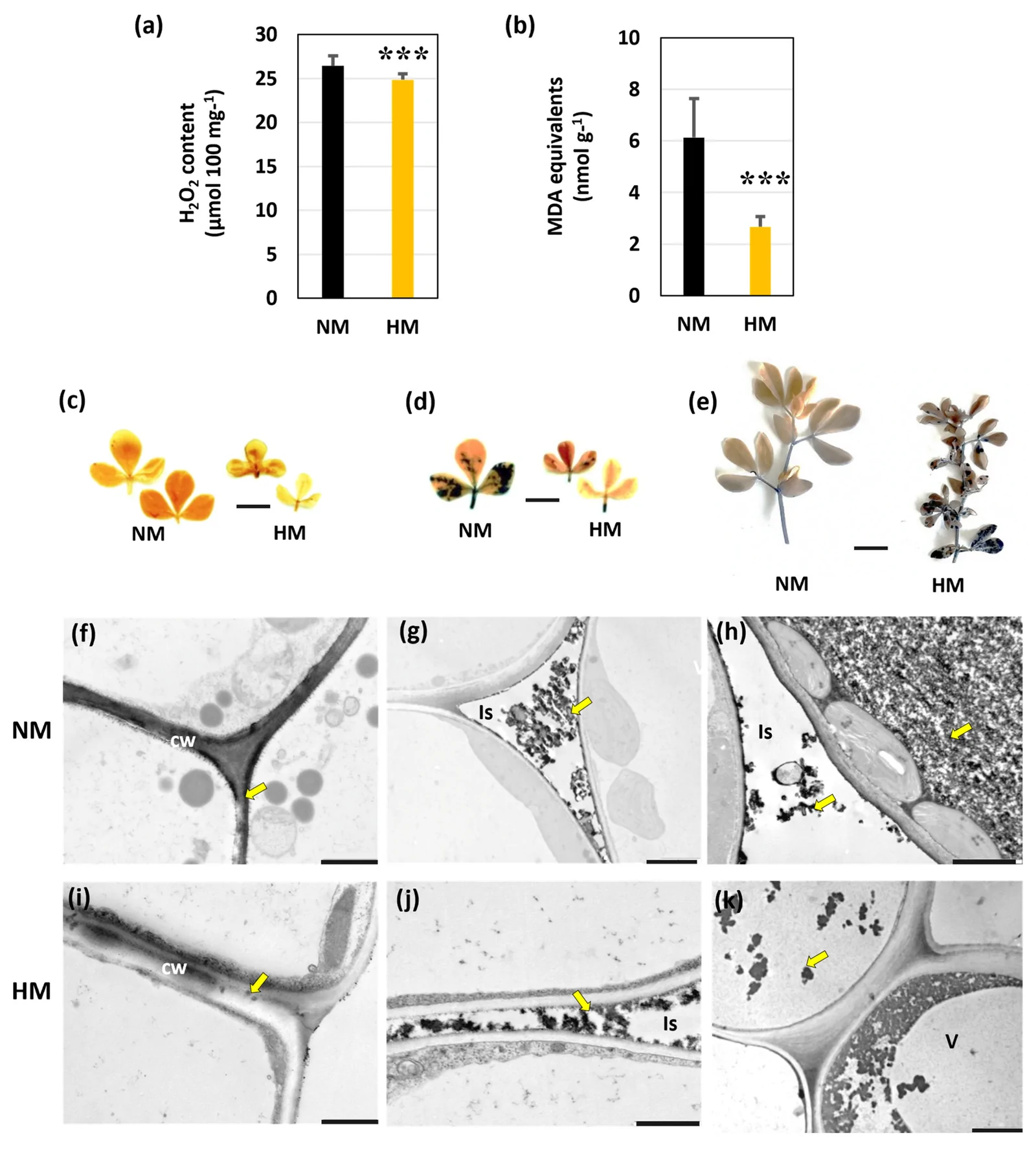
ROS accumulation and oxidative stress level in leaves of control (NM) and calamine (HM) *L. corniculatus* ecotypes. (**a**) Foliar H_2_O_2_ content in NM and HM plants. (**b**) Lipid peroxidation (MDA) in HM and NM shoots. Data are the mean ± SD (n = 3). The stars above the bars indicate significant differences (Tukey’s test, *p* < 0.05). (**c**,**d**) Accumulation of ROS in Lotus leaves by histochemical staining. (**c**) DAB staining for hydrogen peroxide. Brown coloration indicated the presence of H_2_O_2_. (**d**) Reaction with NBT and the formation of blue color indicated the presence of superoxides in leaf tissue. (**e**) Shoots Evans blue staining and dark blue color indicated dead cells. Bars = 1 cm. (**f**–**k**) Hydrogen peroxide localization in leaf mesophyll cells with CeCl_3_. Dark cerium perhydroxide precipitates (arrow) indicate the presence of H_2_O_2_ in the cell walls (**f**,**i**), intercellular spaces (**g**,**j**), and vacuoles (**h**,**k**). Abbreviations: cw—cell wall; Is—intercellular space; v—vacuole. Bars = 1 µm.

As shown in Figure 2, the metallicolous ecotype exhibited a significantly lower accumulation of reactive oxygen species (ROS) in leaves compared to the NM one (Figure 2a,f,g). The HM ecotype showed significantly lower H_2_O_2_ content than the control samples. The quantitative analysis was confirmed by leaf histochemical staining. Diaminobenzidine (DAB) staining for H_2_O_2_ detection and Nitroblue tetrazolium salt (NBT) for superoxide anions also showed less intense HM leaf coloration compared to NM specimens (Figure 2f,g). Hydrogen peroxide was also detected cytochemically via transmission electron microscopy (TEM) through reaction with cerium chloride. The electron-dense precipitates of cerium perhydroxide were typically found near the plasma membranes, in cell walls, in intercellular spaces, and in the vacuoles of mesophyll cells in both ecotypes (Figure 2f–k). Although the distribution patterns were similar in the mesophyll cells of both ecotypes, the precipitates in NM plants were more numerous.

Metal toxicity was assessed by MDA content, a marker of lipid peroxidation and oxidative stress. The HM ecotype exhibited significantly lower MDA levels than the control (Figure 2b). In this study, Evans blue dye exclusion assays indicated localized cell death in response to heavy metal exposure (Figure 2e). Although staining remained negligible in NM shoots (mainly along vascular bounds), the HM ecotype showed significant dye retention in the oldest leaves. This pattern of intense staining suggests that the physiological impact of metal toxicity is most pronounced in old tissues (Figure 2e).

### 3.4. Non-Enzymatic Antioxidants

**Figure 3:**
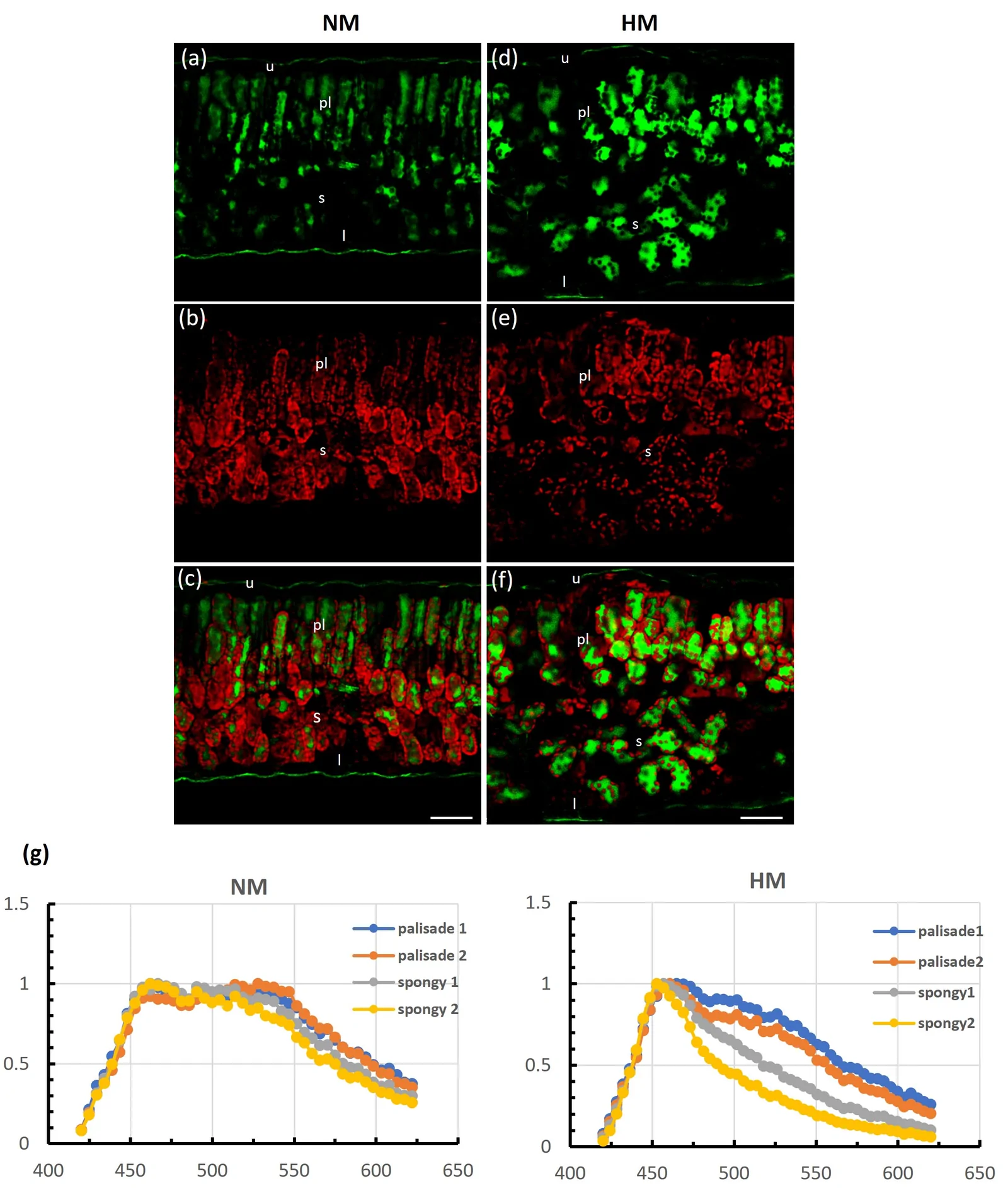
Autofluorescence of phenolics (green channel) and chlorophyll (red channel) is shown on transverse sections through the leaf blade of control (NM) (**a**–**c**) and heap-grown (HM) (**d**–**f**) plants. Note that the vacuoles of palisade and spongy parenchyma cells of plants growing in the heap accumulate increased amounts of phenolics compared to control plants. Excitation 405 nm; emission 420–500 nm phenolics and 660–720 nm chlorophyll. Abbreviations: pl—palisade parenchyma; s—spongy parenchyma, u—upper epidermis; l—lower epidermis. Bars = 50 µm. (**g**) Fluorescence intensity histograms (normalized) of *Lotus* vacuolar phenolics showing the fluorescence profiles during the wavelength scan (λ-Scan) of leaf cross-sections of control (NM) and heap-grown (HM) plants. Excitation wavelength 405 nm; the total range was 200 nm (420–620 nm) with a detection window of 10 nm. Abbreviations: palisade1—first layer of palisade parenchyma adjacent to the upper epidermis; palisade2—second layer of palisade parenchyma; spongy1—first layer of spongy parenchyma; spongy2—layer of spongy parenchyma adjacent to the lower epidermis. The obtained results show differences in the fluorescence emission spectra of vacuolar phenolics. The composition of phenolics in vacuoles changes when plants grow on a heap.

The most prevalent secondary metabolites with a significant physiological role in plants’ antioxidant defence are plant phenolics or polyphenols. Confocal laser scanning microscopy (CLSM) investigation showed that leaves of NM plants accumulate significantly less phenolics in the vacuoles of mesophyll cells than those of the calamine ecotype (HM). Heap-grown plants accumulated higher amounts of phenolics in the vacuoles of palisade and spongy parenchyma cells compared with the control (Figure 3a–f; compare left and right panels).

Figure 3g shows the emission spectra of phenolics in vacuoles of palisade and spongy mesophyll cells. Phenolics in leaves of control NM plants exhibit the same spectral characteristic with a broad range of maximal emission (from 460 to 540 nm), independent of the cell layer or mesophyll type. Leaves of the heap-grown plants (HM) show a narrow (close to 460–470 nm) range of maximal emission.

**Figure 4:**
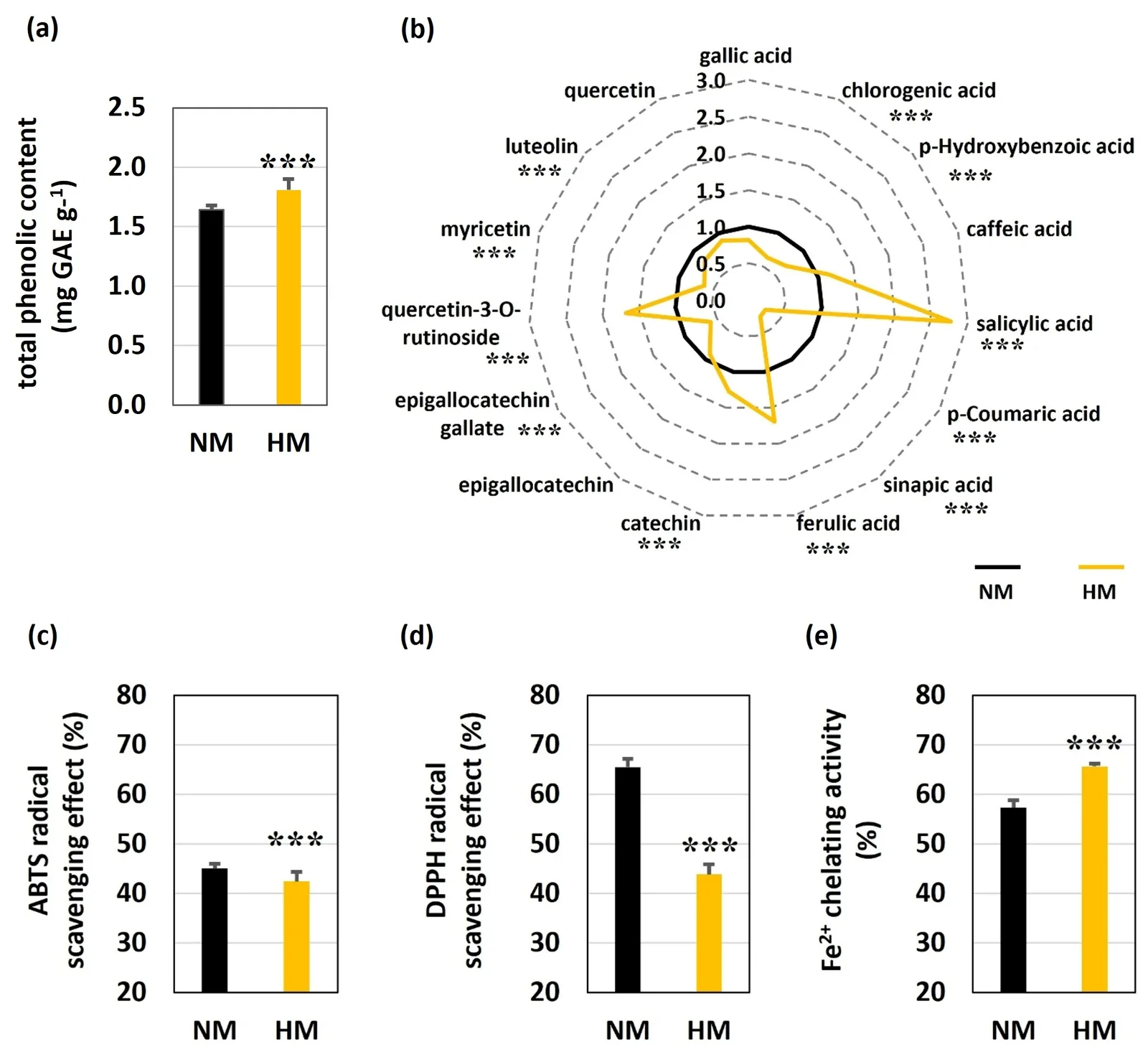
Total phenolic content and profile and antioxidant capacity of control (NM) and calamine (HM) *L. corniculatus* ecotypes. (**a**) Total phenolic content, (**b**) phenolic profile, and spider-plot of selected parameters calculated from the induction curve normalized to the NM. (**c**–**e**) Antioxidant capacity: (**c**) ABTS, (**d**) DPPH, (**e**) FRAP assays (%). Values represent means from at least six plants (n ≥ 6). The stars above the bars indicate significant differences (one-way ANOVA, Tukey’s test, *p* < 0.05).

The confocal microscopy findings on polyphenol presence in *Lotus* leaves were corroborated by spectrophotometric analysis. Figure 4a shows significantly higher total polyphenols levels in the HM shoots compared to the NM. HPLC profiling revealed fifteen polyphenols in the shoots of Lotus samples (Figure 4b). Eight of the phenols were phenolic acids (salicylic, gallic, chlorogenic, p-hydroxybenzoic, sinapic, caffeic, ferulic, and p-coumaric acids), and seven compounds belonged to flavonoids (epigallocatechin gallate, epigallocatechin, catechin, quercetin, quercetin-3-O-rutinoside, myricetin, and luteolin). We observed that the HM samples exhibited a significantly higher content of individual polyphenol compounds, as follows: catechin, quercetin-3-O-rutinoside, and salicylic and ferulic acids (Figure 4b). Interestingly, salicylic acid (SA) and ferulic acid were found to be the predominant compounds in the HM shoots, with approximately higher concentrations compared to NM ones. The HM shoots contained significantly less phenolic acids like chlorogenic, p-hydroxybenzoic, p-coumaric, and sinapic and flavonoids like epigallocatechin gallate, myricetin, and luteolin compared to NM shoots (Figure 4b).

We used three spectrophotometric methods to determine the antioxidant potential in *Lotus* shoots. Interestingly, according to the ABTS and DPPH results (Figure 4c,d), the HM ecotype showed significantly lower scavenging activity (the decrease was about 3% for ABTS and 20% for DPPH) compared to NM, while at the same time, HM plants exhibited the highest ferric-reducing antioxidant ability (FRAP) (65.6% HM and 57.3% NM, respectively) (Figure 4e).

**Figure 5:**
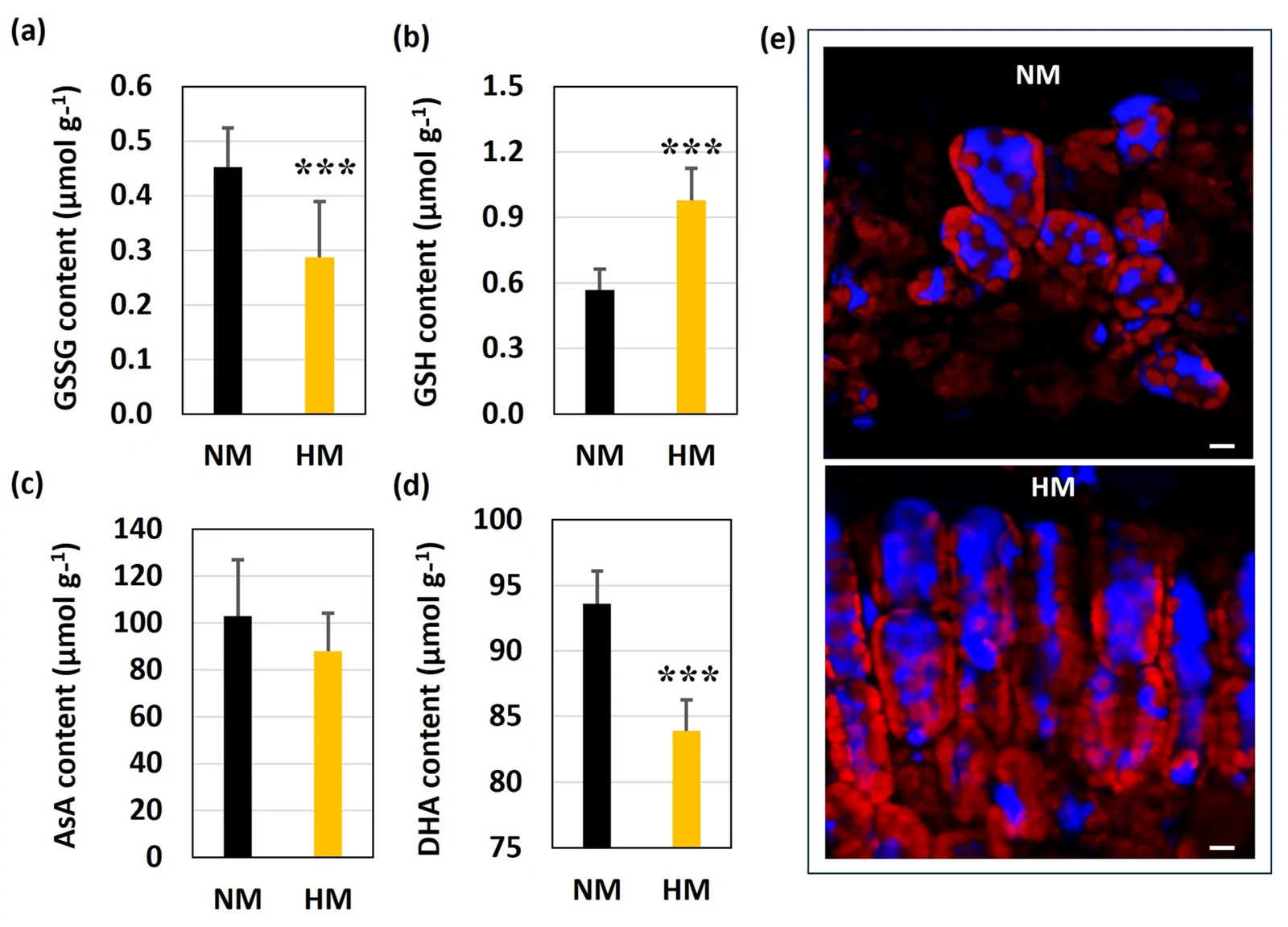
The content of (**a**) oxidized glutathione (GSSG), (**b**) reduced glutathione (GSH), (**c**) ascorbic acid (AsA), and (**d**) dehydroascorbate (DHA) in NM and HM shoots. Data are presented as the mean ± SD of three biological replicates. The stars above the bars indicate significant differences according to one-way ANOVA followed by Tukey’s post hoc test (*p* < 0.05). (**e**) Confocal imaging of GSH conjugation to monochlorobimane (MCB) in the mesophyll of Lotus. Autofluorescence of glutathione S-bimane (GSB) conjugate (blue channel) and chlorophyll (red channel). Visible intense blue fluorescence of GSB conjugates in vacuoles of mesophyll cells and red color of chloroplasts due to chlorophyll autofluorescence in HM and NM leaves. Bars = 5 µm.

Considering non-enzymatic antioxidants, the oxidized (GSSG) and reduced (GSH) glutathione, along with ascorbic acid (AsA) and dehydroascorbate (DHA), which are critical components of the cellular antioxidant defense, were measured together to assess redox status and stress tolerance in Lotus plants. In HM plants exposed to heavy metals, the quantity of GSSG was lower, while the GSH level was about two times higher compared to the NM control (Figure 5a,b). No significant differences in AsA content were observed between ecotypes (Figure 5c). In contrast, the DHA content was lower in HM shoots (Figure 5d).

For the histochemical detection of GSH in leaf tissues, we used the monochlorobimane (MCB) probe, which has been widely used as a fluorescent indicator of GSH [37]. In this method, MCB reacts with GSH to generate a fluorescent GSB conjugate catalyzed by glutathione *S*-transferase (GST). Confocal analysis showed predominant accumulation of the GSB conjugate in vacuoles of palisade and spongy mesophyll cells, with enhanced fluorescence intensity in HM leaves compared with the control (Figure 5e).

### 3.5. Enzymatic Status of Plants

**Figure 6:**
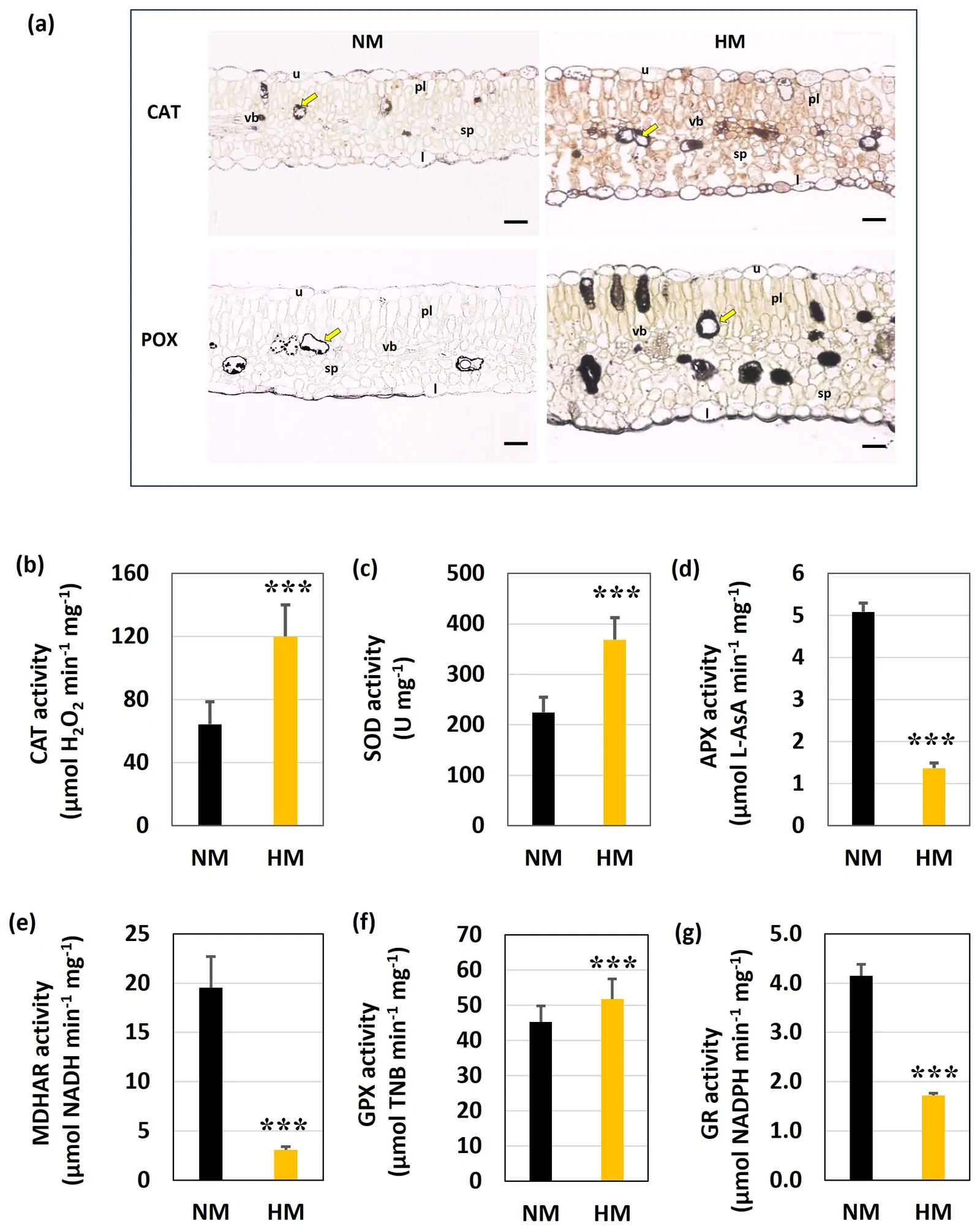
Antioxidant enzyme activities in *L. corniculatus* leaves. (**a**) Histochemical detection of catalase (CAT) and peroxidase (POX) activities in NM leaves. Dark brown coloration indicated enzyme presence. Arrow—phenolic idioblast; pl—palisade parenchyma; sp—spongy parenchyma, u—upper epidermis; l—lower epidermis; vb—vascular bundle. Bars = 50 μm. (**b**–**g**) Antioxidant enzyme activities in *Lotus* shoots, including catalase (CAT) (**b**), superoxide dismutase (SOD) (**c**), ascorbate peroxidase (APX) (**d**), monodehydroascorbate reductase (MDHAR) (**e**), glutathione peroxidase (GPX) (**f**), and glutathione reductase (GR) (**g**). The stars above the bars indicate significant differences at *p* < 0.05 (Tukey’s test).

The histochemical localization of CAT and POD activities in *Lotus* leaves demonstrated that CAT activity was detected in both epidermal layers, in vascular bundles, as well as within the mesophyll, specifically in vacuoles of mesophyll cells that accumulate phenolic compounds (phenolic idioblasts) with more intense coloration in HM leaves (Figure 6a). The POD activity location in both ecotypes was similar to that of those exhibiting CAT activity, but with higher intensity for HM leaves (Figure 6a). Additionally, POD was visualized in both epidermal layers and in vascular bundles.

Statistically significant differences in antioxidant enzyme activities were observed between ecotypes. In HM plants, CAT and SOD activities were almost 2 times higher, while APX activity was 3.3 times lower, comparable with the NM one (Figure 6b–d). The activity of MDHAR and GR was 6.5 times and 2.4 times lower in HM plants, respectively, compared to NM ones (Figure 6e,g). However, GPX activity was significantly increased in HM plants compared with the control (Figure 6f).

## 4. Discussion

The challenging conditions on metal-polluted tailings forced the physiological adaptations that enable plants to colonize these sites based on two main strategies: metal tolerance and metal avoidance [6,38]. Plants known as hyperaccumulators absorb significant amounts of metals, whereas metal excluders minimize toxicity by reducing metal absorption [38,39]. Excluder plants prevent excessive metal translocation to the aerial parts by preferentially sequestering metals in root tissues [39]. *L. corniculatus* is considered a pseudometallophyte, exhibiting increased tolerance to toxic metal levels [4,40,41]. However, the physiological adaptations of this species that spontaneously colonized calamine tailings have never been the subject of research.

Considering the plant’s adaptation to heavy metal presence, the structural and physiological changes in leaves reflect their essential role in plant growth in stressful conditions [6]. Especially for legumes, photosynthetic activity is fundamentally important because it provides high-energy carbon compounds (sucrose/dicarboxylates) necessary to power the energy-intensive fixing process by rhizobia [41,42]. In our previous study on the calamine *A. vulneraria* ecotype and ultramafic *L. corniculatus* ecotype, we demonstrated that prolonged exposure to heavy metals leads to their accumulation, mainly in the roots [3,8]. Additionally, as part of the metal avoidance strategy in calamine *A. vulneraria* leaves, we observed the appearance of a structural barrier with modifications of the leaf apoplast to protect photosynthesis [3]. These findings provide deeper insight into the adaptive strategies of legumes and the mechanisms protecting the photosynthetic apparatus against metal-induced oxidative stress. In calamine Lotus plants, all analyzed heavy metals (Zn, Pb, and Cd) exhibited TF values < 1, suggesting limited root-to-shoot translocation and the protection of aboveground parts from metal toxicity (Table 1). Thus, it was confirmed that L. corniculatus is not able to hyperaccumulate metals, as the average value of the translocation factor was below 1. According to Fernandez et al. [43], the exclusion of heavy metals from aerial tissues represents the predominant defense strategy in non-hyperaccumulating plant species growing on metal-polluted tailings. Thanks to the strategy for avoiding metal accumulation in the shoots, the calamine *Lotus* plants are able to spontaneously colonize the tailings without showing toxicity effects.

### 4.1. Chloroplasts and Photosynthetic Pigments in Contrasting Ecotypes

Despite limited HM transport to the shoots, significant metal accumulation was detected in the aboveground tissues of the calamine ecotype (344.15 mg·kg^−1^ ± 150.6 of Zn; 13.98 ± 3.1 for Pb and 2.79 ± 1.8 for Cd) (Table 1). These metal concentrations in *Lotus* shoots exceed the toxicity levels for most plants [1]. However, TEM analysis showed no toxic effects in mesophyll cells of the HM ecotype. Despite metal stress, the chloroplast of the metallicolous ecotype showed no obvious toxicity symptoms, with intact internal grana and thylakoids and small plastoglobules. Thus, no starch grains were observed in spongy or palisade parenchyma cells (Figure 1). The absence of starch in chloroplasts under heavy metal exposure may reflect an intensified allocation of assimilates toward the symbiotic interactions, the synthesis of metal-chelating compounds, enhanced antioxidant metabolism (including the ascorbate–glutathione cycle), and/or cellular repair processes [44]. The high energy requirements associated with metal detoxification and maintenance of tolerance mechanisms may impose growth penalties, including reduced biomass accumulation, dwarfism, and impaired shoot development in metal-tolerant ecotypes relative to sensitive populations [45].

It is well documented that exposure to heavy metals can reduce photosynthetic pigments, thereby affecting photosynthesis [12]. In this study, *L. corniculatus* ecotypes differed in photosynthetic pigment content and composition. The metallicolous plants showed lower total chlorophyll content, resulting from a decrease in chl *b*. Similar pigment reduction was observed in metal-tolerant non-legumes from metal-polluted wastes [46]. Taking into account the fact that plants growing on the tailings, in addition to high metal contamination, also have to cope with high insolation, the observed decrease in chl *b* might be a way to avoid photooxidative damage and cell death. Sato et al. [47] showed that under high-light conditions, reduced chl b is a key adaptive mechanism in plants, allowing them to manage excess energy, prevent the plant from absorbing more light than it can process, and reduce photodamage.

Moreover, metallicolous plants showed a lower content of total carotenoids as well as total xanthophylls and carotenes than NM plants. At the same time, a significantly elevated de-epoxidation state of xanthophyll-cycle pigments was observed in the HM ecotype, indicating enhanced photoprotective capacity [48]. The results indicate a minor role of carotenoids as antioxidants in plants growing on calamine tailings. Additionally, the reduced chl *b* synthesis, combined with the unchanged chloroplast ultrastructure and higher DES value, indicates an effective mechanism of photosynthetic apparatus protection in HM plants and stress acclimation.

### 4.2. Oxidative Stress Level in Tested Ecotypes

Reactive oxygen species function as key signaling molecules involved in the regulation of plant responses to a wide range of stimuli; however, their role transitions from signaling to damage when the rate of production outpaces the scavenging capacity of the antioxidant system [41]. Heavy metal excess induces ROS accumulation, causing systemic oxidative stress and leading to deleterious modifications to DNA, proteins, and lipid membranes, and, in severe cases, programmed cell death (PCD). In this study, despite the high mobility of metals into the shoots, the ROS and malondialdehyde (MDA) levels were low in calamine plants, suggesting the plant’s capacity for metal stress neutralization (Figure 2). The metallicolous ecotype exhibited decreased generation of ROS in shoots, which may indicate the more efficient mitigation of oxidative stress in this ecotype. Reduced H_2_O_2_ accumulation in HM leaves was confirmed by CeCl_3_ staining and TEM analysis, which showed low peroxide presence in the apoplast and vacuoles of HM mesophyll cells. Prior research indicates that a defining trait of metal-tolerant species is the maintenance of suppressed ROS levels, preventing the systemic oxidative damage typically seen in sensitive genotypes [48,49]. In addition, the marked accumulation of Evans blue in old leaves suggests that these tissues underwent localized necrosis (Figure 2e). The intensified Evans blue staining observed in the older leaves of HM plants is consistent with the established pattern of heavy metal sequestration in senescing tissues [50]. This preferential accumulation in older foliage is often interpreted as a protective strategy that shields younger, more photosynthetically productive leaves from metal-induced oxidative impairment. Certain tailings plants, including *A. vulneraria* and *Biscutella laevigata*, translocate excess metals to aging leaves and organs for seasonal metal removal [51]. Our research demonstrated that in metallicolous *Lotus* plants, detoxification strategies mainly involve the sequestration of metals into inactive compartments and the regulation of ROS levels.

### 4.3. Antioxidant System Activity

In response to metal-induced ROS accumulation, plants activate sophisticated antioxidant defense systems containing non-enzymatic antioxidants. Polyphenols represent a major class of secondary metabolites involved in ROS scavenging, the regulation of membrane fluidity, and metal ion chelation [16,52]. Hence, the phenolic content in plant tissues serves as a useful indicator of plant stress tolerance. In our study, CLSM analysis revealed higher polyphenol accumulation in the calamine ecotype compared to the NM ecotype (Figure 3 and Figure 4). CLSM studies showed a more intense accumulation of phenolic compounds in the vacuoles of the leaf mesophyll of calamine plants (Figure 3). Furthermore, a comparative analysis of phenolic emission spectra revealed significant spectral modulations in the metalliferous ecotype compared to control specimens. The narrow maximum emission range characteristic of plants grown on heaps may result from the dominance of phenolic compounds, which exhibit a maximum fluorescence near 460 nm. Similarly, analytical studies revealed a significant increase in the total phenolic content in the calamine plants compared to the control ones (Figure 4). HPLC analysis showed that the phenolic composition of HM shoots differed significantly from that of NM plants (Figure 4b). Calamine shoots showed increased accumulation of catechin, quercetin-3-O-rutinoside, salicylic acid, and ferulic acid, whereas they showed lower levels of chlorogenic, p-hydroxybenzoic, p-coumaric, and sinapic acids, as well as epigallocatechin gallate, myricetin, and luteolin. The physiological role of both phenolic acids and flavonoids is closely linked to plant responses to stress factors. The observed increase in catechin in the HM ecotype might suggest active metal ions chelation, as they form non-toxic metal-catechin complexes, which prevent the ions from reaching sensitive organelles like chloroplasts and mitochondria [53]. An increase in rutin (quercetin-3-O-rutinoside) content was also observed in metal-treated *Zea mays* plants, where this flavonoid may contribute to antioxidant protection [53]. The increased accumulation of ferulic acid detected in the HM ecotype may indicate enhanced antioxidative capacity and potential metal-chelating activity. Ferulic acid is known for its diverse biological functions, including antioxidant properties, metal ion chelation, and the regulation of enzyme activity. It is commonly present as a covalently bound component of plant cell wall polymers and acts as an efficient UV absorber [54]. Additionally, HM plants showed higher salicylic acid content compared to the NM ecotype. Salicylic acid, a phenolic phytohormone, is an important regulator of plant physiological processes and stress responses. Under heavy metal stress conditions, it reduces metal uptake, interacts with other hormones, and enhances antioxidant defenses, thereby mitigating metal toxicity [55,56]. The observed decrease in some phenolic concentrations in the HM ecotype might suggest their active consumption during the defense responses [16]. Coumaric acid is used as an intermediate substrate for the biosynthesis of further phenylpropanoid acids and flavonoids [57] and shows high affinity for metal complexation [20,52]. Furthermore, phenolic acids participate in lignin formation, strengthening cell walls under heavy metal stress [58]. Phenolic compounds have a high tendency to chelate metals; thus, the roots of many plants exposed to heavy metals exude high levels of phenolics [16,20,49,52]. It is another piece of evidence that the lack of oxidative stress in HM plants is connected with reduced metal toxicity through metal chelation. The results obtained by the HPLC method were reflected in the CLSM analysis. Our results show that ferulic acid and catechin, which fluoresce blue, increased in HM plants, while quercetin, which fluoresces green, decreased [58]. Although the rutin content increased significantly in the calamine plants and this compound shows blue fluorescence, it has a low quantum yield [59]; therefore, it probably does not have a significant effect on the intensity of blue fluorescence. Strikingly, under stress conditions induced by growth on metal-polluted heaps, individual mesophyll cell layers of HM plants exhibit different emission characteristics, which is pronounced in the spongy parenchyma, particularly the spongy2-layer (see Figure 3). Similar high phenolics vacuolar in mesophyll cells accumulation was observed by the cytochemical localization of CAT and POX, suggesting their involvement in the response to metal stress (Figure 6a). It seems that in leaves of the HM ecotype, different mesophyll layers use different survival strategies, where lower mesophyll layers may be primary sites for metal sequestration. A similar strategy was observed in the hyperaccumulator *Arabidopsis halleri,* where the plant effectively “dumps” toxic zinc or cadmium into the cells of the spongy2-layer to protect the rest of the leaf [60].

In this study, three spectrophotometric methods were used to determine antioxidant potential in *Lotus* plants. Despite the high total phenolic content, phenols occurring in shoots of the calamine ecotype had a rather low ability to scavenge DPPH and to engage in ABTS+ radical scavenging. This lower-than-expected antioxidant activity is because this group of phenols might be specialized for metal chelation rather than general radical scavenging [15]. The HM ecotype consistently demonstrated high total phenolic values, coupled with superior antioxidant capacity, as confirmed by FRAP assays. Higher FRAP activity in the HM ecotype indicates a more effective reducing capacity associated with its specific polyphenol composition.

In addition to phenolic compounds under metal stress, reduced glutathione (GSH) participates in ROS detoxification and is a key metal chelator [14]. As an antioxidant, GSH reduces hydrogen peroxide by undergoing an oxidation-reduction cycle with glutathione peroxidase (GPX), glutathione reductase (GR), and ascorbate (AsA) known as the AsA-GSH cycle [61]. Metal stress induces substantial alterations in the regulation of GSH biosynthesis [62]. The presence of heavy metal ions activates the phytochelatin detoxification system, which includes low-molecular compounds such as cysteine, glutathione, and its homologues, phytochelatins, as well as enzymes of glutathione and phytochelatin synthesis [63,64]. The adaptation of calamine *Lotus* plants to metal stress was accompanied by increased leaf GSH accumulation, indicating its dual function in metal chelation and ROS scavenging. However, the high GSH content in the HM ecotype was correlated with a simultaneous decrease in its oxidized form. Interestingly, we showed low GR activity in HM plants, which takes part in GSH regeneration. Since GR is responsible for the reduction in oxidized glutathione (GSSG) to regenerate GSH, the predominance of GSH over GSSG, with the reduction in GR activity, may result from compensatory mechanisms such as enhanced de novo GSH synthesis or GSSG removal [63,64]. AsA is a central antioxidant molecule in plant cells, playing an essential role in the ascorbate–glutathione cycle and redox homeostasis. Monodehydroascorbate reductase (MDAR) is the enzymatic component of this cycle that regenerates reduced AsA. Calamine *Lotus* plants show low oxidative or reduced AsA content and low MDHAR activity, indicating low oxidative stress and no need to run the AsA-GSH cycle (Figure 5 and Figure 6). The cytoplasmic GSH can be labelled with monochlorobimane (MCB). MCB enables the visualization of GSH-dependent xenobiotic detoxification pathways in plant cells, as GSH conjugates are transported and sequestered within vacuoles [30]. The elevated level of GSH–bimane conjugates detected in vacuoles of mesophyll in HM plants suggests intensified GSH-dependent sequestration processes, potentially contributing to metal complexation and vacuolar detoxification in calamine *Lotus* plants. A similar strategy of metal detoxication was observed in hyperaccumulators [65,66]. GSH functions as a key metal chelator, forming metal-GSH complexes that facilitate their sequestration and excretion. The glutathione S-transferases (GSTs) catalyze this metal conjugation. GSH is also a precursor of metal-binding phytochelatins (PCs). Metal ions that enter the cytoplasm are chelated by glutathione or PCs, forming low-molecular-weight complexes, which are then transported to the vacuole, where more stable complexes are formed [63,64,65,66]. Taking into account our findings, in calamine Lotus plants, increasing GSH concentrations contribute to minimizing the metal stress by metal chelation, and/or the protection of SH groups in proteins, rather than ROS scavenging. Therefore, it can be suggested that metal tolerance in this ecotype is associated with the activation of efficient metal detoxification mechanisms, including glutathione and metal-binding phytochelatins, although this finding requires further study.

The enhancement of antioxidant enzyme activity is a crucial mechanism for mitigating cellular damage caused by metal-induced lipid peroxidation, thereby strengthening plant stress resistance [67]. In the calamine Lotus ecotype, the antioxidant defense is primarily driven by the heightened activity of superoxide dismutase (SOD), peroxidase (POX), catalase (CAT), and glutathione peroxidase (GPX) (Figure 6). These enzymes function as the central protective system, mitigating the oxidative damage typically induced by heavy metal exposure [67]. Plant POXs comprise several forms, including soluble, cell wall-bound, apoplastic, and vacuolar enzymes. Among them, cell wall-bound POXs have been widely associated with lignin deposition and the regulation of cell wall structure. Soluble, apoplastic POXs can scavenge peroxide, cooperating with phenolics and AsA [10,67]. This was in agreement with our histochemical detection of POX activity in Lotus leaves, where we showed its higher activity in HM plants, in epidermis, in vascular bundles, and in mesophyll cells, specifically in phenolic-containing cells (Figure 6). Thus, GPXs are non-heme peroxidases which participate both in the maintenance of membrane integrity and in the H_2_O_2_ homeostasis, especially under metal stress [68]. In this study, observed in calamine plants, the elevated GPX activity and reduced APX activity suggest that metal stress induces a shift in antioxidant defense. It has been demonstrated that GPX can prevent excessive ROS accumulation and limit lipid peroxidation when APX activity is reduced [69]. Additionally, CAT breaks down hydrogen peroxide into water and oxygen. While metal toxicity can inhibit CAT, tolerant plant species often show elevated, stable CAT activity in leaves and roots [10,67,70]. In this study, increased CAT activity in HM shoots resulted in the low accumulation of peroxide that was visualized by DAB staining (Figure 2 and Figure 6). Thus, SOD is responsible for the enzymatic conversion of superoxide radicals into hydrogen peroxide through the dismutation reaction. Increased SOD activity in HM shoots is compatible with NBT staining (Figure 2), which showed reduced superoxide anions accumulation. Several studies confirmed our observation that the treatment of heavy metal increases the activities of SOD, CAT, and POX [10,67,70]. These results suggest that the induction of CAT, SOD, POX, and GPX provides an effective enzymatic shield against oxidative burst in the *Lotus* calamine ecotype.

**Figure 7:**
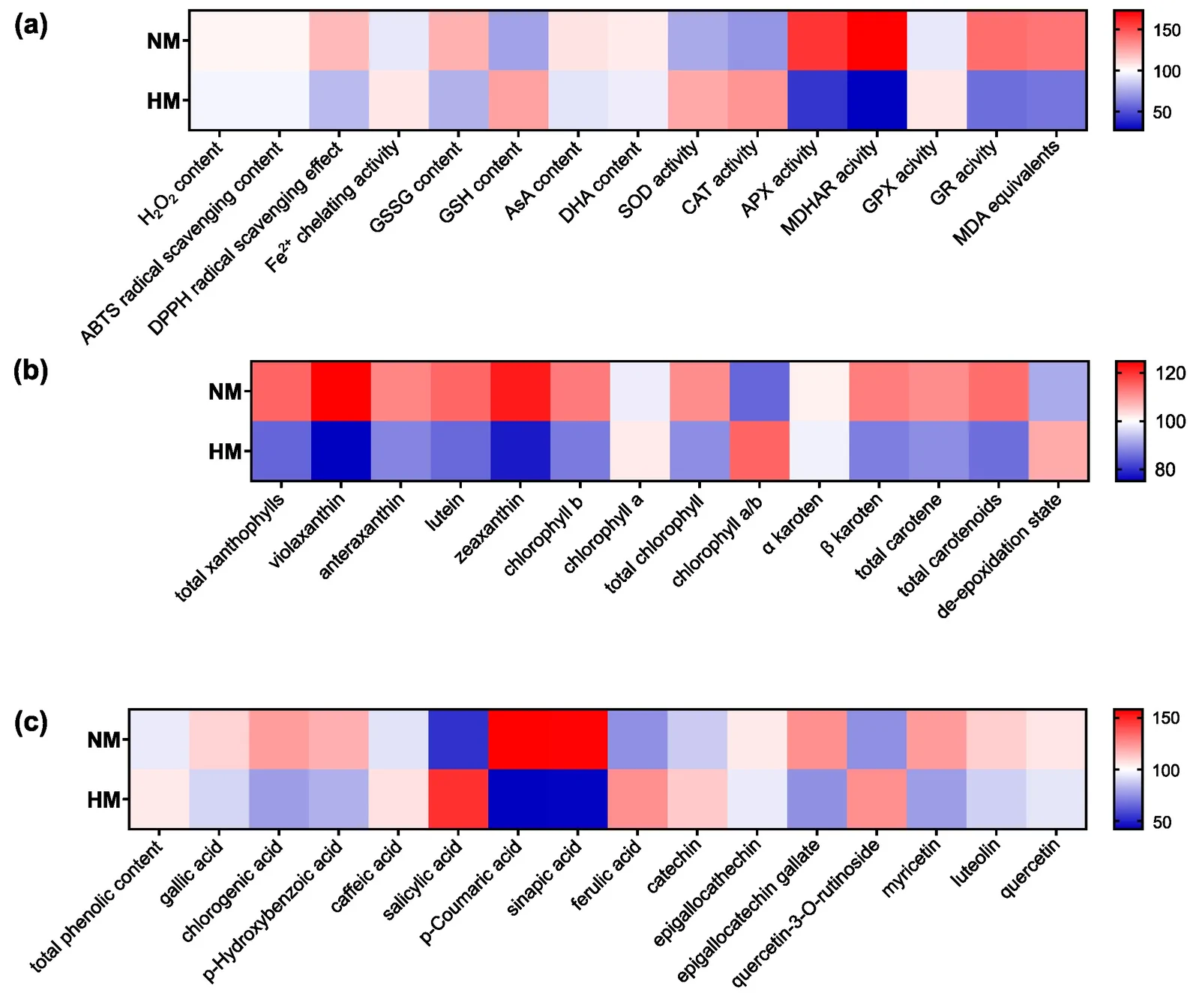
Heatmap representation of physiological and biochemical parameters in NM and HM ecotypes. The values for each parameter were scaled relative to the mean of both ecotypes, with the mean set to 100. Parameters are grouped into (**a**) oxidative stress and antioxidant system, (**b**) photosynthetic pigments, and (**c**) phenolic compounds.

The integrative heatmap analysis (Figure 7) highlights a clear divergence in adaptive strategies between ecotypes, revealing coordinated clustering of physiological and biochemical traits. The HM ecotype is characterized by reduced oxidative stress and lipid peroxidation, alongside elevated antioxidant enzyme activity (SOD, CAT, GPX) and the increased accumulation of glutathione and selected phenolics. In contrast, enzymes associated with the AsA–GSH cycle show lower activity, indicating a reduced dependence on classical ROS-scavenging pathways. Phenolic compounds contribute mainly to metal chelation rather than direct antioxidant defense, while changes in pigment composition suggest enhanced photoprotection. Together, these patterns indicate that the adaptation of *L. corniculatus* to metal-contaminated environments is driven by efficient metal detoxification and the prevention of oxidative stress rather than its subsequent scavenging.

## 5. Conclusions

The results showed that calamine *Lotus* plants exhibit effective adaptation to long-term metal exposure by keeping metals away from metabolically active compartments and strengthening antioxidant defenses. These coordinated mechanisms reduce oxidative stress and contribute to the enhanced metal tolerance of calamine plants.

## Figures and Tables

**Table 1:** Content of selected metals in shoots and roots of *L. corniculatus* NM and HM ecotypes (mg·kg^−1^ dry matter).

Metal		NM			HM	
	Shoot	Root	TF	Shoot	Root	TF
**Zn**	18.3 ± 1.04 b	20.6 ± 2.61 b	0.8	344.1 ± 150.6 a	1042 ± 223.8 a	0.3
**Pb**	1.8 ± 0.62 b	3.1 ± 0.28 b	0.5	13.9 ± 3.1 a	71.2 ± 11.4 a	0.2
**Cd**	0.04 ± 0.01 b	0.2 ±0.24 b	0.2	2.8 ± 1.8 a	14.2 ± 6.3 a	0.2

TF—translocation factor. Data are means ± SD. Different letters indicate significant differences (one-way ANOVA, Tukey’s test, *p* < 0.05).

## Data Availability

The experimental data supporting the findings of this study are available from the corresponding author upon reasonable request.

## Abbreviations

The following abbreviations are used in this manuscript:

HM	Metal-contaminated ecotype
NM	Non-metal-contaminated ecotype
SOD	Superoxide dismutase
CAT	Catalase
POX	Peroxidase
AsA	Ascorbate
GPX	Glutathione peroxidase
GSH	Glutathione
GR	Glutathione reductase
MDA	Malondialdehyde
MDAR	Monodehydroascorbate reductase
ROS	Reactive Oxygen Species
TEM	Transmission Electron Microscope
